# Suffering while resigning to an unacceptable violation of dignity

**DOI:** 10.1177/09697330231209295

**Published:** 2023-10-22

**Authors:** Trude A Hartviksen, Jessica Aspfors, Lisbeth Uhrenfeldt

**Affiliations:** 8016 UiT, The Arctic University of Norway; The Muncipality of Vestvågøy; 1786 Nord University; 1040 Åbo Akademi University; 91921 Kolding Hospital; 555154 Southern Danish University

**Keywords:** Relatives, nursing homes, suffering, undignified care, focus groups

## Abstract

**Background:**

The interaction of health personnel with relatives is linked to the quality of care results in nursing homes. However, there is limited knowledge of how relatives perceive being an integral part of the nursing home context. This secondary analysis has its starting point in an ethical concern about relatives’ experiences in a previous study.

**Aim:**

To critically discuss relatives’ experiences of suffering when their next of kin live in a nursing home in a rural arctic context.

**Research Design, Participants and Context:**

The critical hermeneutic stance is informed by Habermas. The secondary analysis is conducted on original data from five semi-structured focus groups with 18 relatives of residents of two nursing homes in a rural part of Norway. The theoretical framework concerning dignity, well-being, and suffering, as developed by Galvin and Todres, contrasts the analysis.

**Ethical Considerations:**

The study followed the principles of the Helsinki Declaration. It was approved by the Norwegian Center for Research Data (NSD) (reg. no. 993360).

**Findings:**

The main theme of this study is: suffering while resigning to an unacceptable violation of dignity. This theme is deepened by two subthemes: (a) suffering while adapting to a relationship of dependence and (b) suffering while accepting the unacceptable.

**Conclusions:**

Relatives experience suffering as a cross-pressure in their struggle to interact responsibly with health personnel in nursing homes. This may have a negative outcome, where relatives end up adapting to being silent witnesses to missed care and a violation of dignity.

## Introduction

The involvement of relatives is recognized as an essential approach by health authorities, supported by quality benefits and human rights considerations.^1^ Health personnel’s close interaction with relatives is linked to the quality of care results in nursing homes.^2,3^ However, studies involving relatives of nursing home residents predominantly depict their negative experiences. When relatives’ expectations are not met, it is shown to lead to suffering.^4^ Existing studies are referred to as primarily descriptive and relatively atheoretical. Consequently, there is limited knowledge of the underlying stressors behind the described suffering and how it can be mitigated. In particular, there is a significant knowledge gap regarding these sociodemographic dynamics in rural contexts.^2^

## Background

In this study, the term relatives is broadly understood as significant others, next of kin, family members or loved ones, to older persons living in nursing homes. A nursing home is recognized as an institution that offers housing, support and care to frail older persons who are unable to live independently.^5^ The reasons why a person may become a nursing home resident are described as illness, injury or normal aging. Relatives have depicted it as necessitated by a “life crisis.” This crisis includes a loss of functioning and may be socially related to the relatives’ fear and insecurity about what could happen in the home.^6^

Several reviews indicate that relatives continue as central caregivers for residents in nursing homes.^2,6,7^ However, this active involvement applies to relatives in rural areas to a lesser extent.^2^ In a critical interpretive synthesis conducted by Puurveen et al.,^8^ various roles and responsibilities of relatives are identified, emphasizing their foundation in the family-resident relationship, influence from family-health personnel relationships, and impact from broader sociopolitical factors. Pulst et al.^7^ describe how health personnel in nursing homes confront relatives to make treatment and transfer decisions. Curtis et al.^9^ show how trusting relationships between health personnel, residents and relatives, including good communication and ethical practice, improve the quality of emergency care for residents.

When relatives’ expectations are met, they describe engagement, inclusion, and a good atmosphere in the nursing home. However, when their expectations are not met, suffering is portrayed, described as a sense of powerlessness, distrust, and guilt.^4^ This study is supported by Galvin and Todres’^10^ existential theoretical framework, where the relationship between well-being and suffering is explained as a continuum with 18 variations. These are distributed in six domains: spatiality, temporality, intersubjectivity, mood, identity, and embodiment.^10^ In a previous study,^11^ we demonstrated how the experiences of suffering among relatives of nursing home residents can be related to all six domains.

Galvin and Todres^12^ describe dignified care as the preservation of a person’s honor and values while acknowledging their vulnerability. However, the Francis Report served as a wake-up call for the National Health Service in the United Kingdom, revealing severe missed care.^13^ Missed care refers to the omission or delay of any aspect of patient care. This presents ethical dilemmas for nurses, as it contradicts their professional and moral principles.^14^

Indignity in the care of older persons arises when health personnel exhibit unethical attitudes, for example, by creating feelings of powerlessness, abandonment, being disregarded or not being taken seriously.^15^ The prevalence of abuse and neglect of older persons in institutional settings is high.^16^ There is substantial evidence linking missed care to indignity in nursing homes.^17–19^ Due to the complexity of their care needs, a special risk exists for residents with chronic health conditions, such as dementia or other degenerative illnesses,^20,21^ residents with a loss of vision or hearing, or those without relatives to support their need for care and social interaction.^21^

Although the importance of relatives’ involvement in nursing homes is widely recognized, there is a need for additional research to fully understand the communication dynamics between relatives and health personnel in various settings.^22^ It is essential to acknowledge the capacities and limitations of relatives in order to gain a comprehensive understanding of this issue.^8^ This study aims to explore these aspects specifically in relation to relatives’ experiences of suffering, using the theoretical framework developed by Galvin and Todres^10^ to elaborate on this concept.

## Aim

This study aims to critically discuss relatives’ experiences of suffering when their next of kin live in a nursing home in a rural arctic context.

## Methods

### Research design

Habermas^23,24^ informs the critical hermeneutic stance in all research phases of this study. Galvin and Todres^10,12^ illuminate the theoretical framework of dignity, well-being, and suffering. This secondary analysis^25^ includes all data from five semi-structured focus groups conducted in a previous research project, including 18 relatives of residents of two nursing homes in a rural part of Norway.^11,26,27^

The critical hermeneutic stance implies in this study that, in accordance to Habermas,^23^ the participants’ lifeworlds, are pre-understood as colonized by the system. This secondary analysis was initiated based on the authors’ concern in a previous study^26^ about the participants’ experiences of suffering when being relatives in nursing homes. Further, critical reflection balances the research process. This involves searching for contrasts and accentuating theoretical statements that represent changeable dependent relationships.^23^

### Participants and context

All relatives, representing 95 residents from two publicly financed nursing homes in rural Norway, were invited to participate in the research project, without further inclusion or exclusion criteria. Nursing home A provided housing, support, and care for 50 residents diagnosed with dementia. The 45 residents in nursing home B had extended care needs due to various diagnoses. Among the invited relatives of the 95 residents, 18 participated. Two were sisters, and two were mother and son. The participants included 11 women and seven men. Of these, 11 relatives were adult children, six were spouses, and one was a son-in-law. Their ages ranged from 34 to 90 years old. The participants’ experience of being relatives of nursing home residents concerned a minimum of 1 year and a maximum of 7 years.

### Ethical considerations

The study followed the principles of the Helsinki Declaration. It was found not to require application by the Regional Committee for Medical and Health Research Ethics (REK)^28^ (reg. no. 2018/1905) and was registered at the Norwegian Center for Research Data (NSD)^29^ (reg. no. 993360). Before data collection, the participants were informed orally and in writing about the study’s aim, protection of their confidentiality, and their personal right to withdraw at any time in the process.^29^ The written information and consent form are disseminated.^27^ None of the participants withdrew during the study. Their consent included the possibility of the empirical data being reused for other studies of nursing home quality within a 5-year period. Throughout this secondary analysis, steps were taken to fulfill established criteria for trustworthiness, including credibility, dependability, confirmability, and transferability.^27^

### Data collection

Data were collected through five focus groups in closed settings: two in a meeting room in one of the nursing homes and three in the other. A written information and consent form was distributed to each relative in advance by middle managers at the nursing homes. This invitation explained the aim of the study and presented the authors with photos and text.^27^ Qualitative semi-structured research interviews guided the dialog in the focus groups, offering enough time for an open dialog between the participants, as well as the data collector, who also had the role of moderator.^30^

The semi-structured interview guides are disseminated.^27^ The open-ended questions were framed around participants’ characteristics, nursing home quality, leadership development, and experienced changes in nursing home quality after leadership development. The interviews lasted approximately one and a half hours and were audio-recorded and transcribed verbatim, together with notes from an observer. The observer supported the interviewer with additional data on the participants’ reactions and interactions.^30^ When all three authors considered that the themes that emerged from the data were repeated rather than developed, data saturation was considered,^31^ and no further focus groups were added.

### Data analysis

Initially, the transcribed data underwent content analysis by the first author, searching for latent content by constructing themes through coding the relatives’ perspectives.^32,33^ All three authors participated in critical reflection and discussion, where the main theme was constructed. After identifying the main theme, the analysis was supplied with a stepwise thematic analysis^34^ by the first author, through which we found the subthemes that revealed deeper insights into the relatives’ suffering. The last author progressed the analysis through a constant critical analysis of data, based on the understanding of dignity, well-being, and suffering, as presented in the framework of Galvin and Todres.^10,12^ The second author mainly focused on the stringency of the analysis from aim to findings.

## Findings

This study has one main theme: suffering while resigning to an unacceptable violation of dignity. The main theme is deepened by two subthemes: (a) suffering while adapting to a relationship of dependence and (b) suffering while accepting the unacceptable.

### Suffering while resigning to an unacceptable violation of dignity

The main theme, suffering while resigning to an unacceptable violation of dignity, provides evidence of how relatives in nursing homes described suffering when they chose not to intervene, despite their perception that the dignity of the residents was threatened. The participants described how they saw themselves as a “fly on the wall” and gave a number of examples of incidents where they had observed undignified care. Instead of intervening, the relatives elaborated on how they as part of their approach to acceptance tried to understand, explain, and accept why such incidents occurred. The participating relatives amplified that their relationship to the nursing home was to be described as dependent, as they were aware that the nursing home was the only option for their next of kin. They considered it impossible for the resident to continue living at home. Rather than questioning the nursing home practice, they therefore tried to adapt to the institutionalized rules. However, they experienced the rules as inconsistent and as individually interpreted by the health personnel. They described the process of accepting missed care as demanding and burdensome for their conscience.

### Suffering while adapting to a relationship of dependence

The subtheme, suffering while adapting to a relationship of dependence, emerged from the findings, as relatives shared their experiences of asserting dignity as a formidable task that necessitated both adaptation to the institutional constraints and at the same time safeguarding the essence of a family relationship. Some relatives described entering the nursing home due to a gradual decline in their next of kin’s physical and cognitive functioning, reaching a point where living at home was no longer feasible. For others, the transition was more abrupt, for example, as a consequence of the resident having suffered a stroke. Regardless of the gradual or abrupt nature of the transition, all participating relatives described a challenging process characterized by feelings of insecurity, vulnerability, and grief. They had to come to terms with the nursing home as the only viable option and described this as a relationship where their dependence required them to adapt to institutional settings more than it ever could be considered as visiting the resident’s home. Participant 2, a daughter, exemplified this in the fourth focus group:

*…it will never become a home, no, never... and this will never change... a home, then you come to visit people and then you can sit down together and eat a meal and enjoy yourself. However, there (at the nursing home), you are chased into the room…no, it is thus far from a home…a yellow note has appeared that says that we must sit in the rooms when we visit. That is not a home…*

The participating relatives described trying to create a home atmosphere when visiting the nursing home. They emphasized the importance of socializing around a coffee table or during mealtimes, as it allowed the resident to maintain their dignity, mirroring how they would have hosted guests at home. The participants described visiting the residents frequently. Several visited daily, some visited several times a day, and some visited weekly. However, the relatives’ descriptions of attempts to create a home atmosphere were in contrast to their critical discussions of visits adapted to what they described as a hostile environment. Several of the participants depicted feeling unwelcome and shared the experience of encountering different rules and restrictions from visit to visit, both verbally and on written messages at walls. They discussed how health personnel had told them that they were a disturbance if they visited the resident in the communal areas; they were asked to lower their voices, to not enter the kitchen and to take the resident into their room.

On encountering these restrictions, the participants explained how they mainly chose not to question them but rather tried to adapt when necessary. The rules were experienced as being enforced differently, depending on which health personnel were present at work. Participant 3, a daughter, explained in the first focus group:

*... and then it is like, yes, it is so uneasy for the other residents, because we talk a little... yes, we have been told that. It is clear that when they are at work those who have given notice, then you dare not to do anything but... just fiddle into the room...*

This pronounced resignation leads further to the data that forms the second subtheme provided by these findings.

### Suffering while accepting the unacceptable

The findings that constitute the second subtheme, suffering while accepting the unacceptable, show the accounts provided by participating relatives regarding their continuous observation of the residents’ dignity and assessment of their well-being in the nursing home. The participants reflected on various factors, such as the quality of food, the duration of night fasting, changes in the resident’s weight, and other everyday assessments. These descriptions encompassed both the relatives’ experiences of dignity and instances of indignity arising from missed care and evoked feelings of suffering. The relatives critically reflected on what they described as unacceptable situations they personally witnessed, as well as those they were informed of through conversations with health personnel working in the nursing homes.

Among these observations, the relatives explained how they observed the residents’ life to lack content and activity, that there were no offers of individual meaningful activities and that those residents who required help with relocation were less often invited to participate in group activities. It was described how residents were put to bed after lunch to give the health personnel some spare time, and how the residents were put to bed for the night at 18:00–18:30, which was related to how the health personnel prepared the unit to be ready (calm) before the night shift arrived. The relatives gave examples of the residents’ lack of self-determination and observations of the use of coercion. Three individual relatives described how they had suffered silently while they observed how the lack of exercise after fractures led to functional decline for the resident.

The participants explained why, despite suffering while observing it, they attempted to accept an unacceptable practice. The explanations were a lack of information or communication, personnel shortage, the municipality’s financial priorities, or, as participant 16, a wife, explained in focus group 3, how health personnel have a challenging job:

*…when we talk about indignity... in nursing home A, it is not the easiest to care for people, we know that, but still they manage to keep clothes clean, and, yes, my husband has been difficult to care for, it is difficult to dress him, currently it’s a little easier because he’s more compliant…*

Relatives’ descriptions of the challenging nature of the health personnel’s job and that they nevertheless went the extra mile for the residents was a recurring topic in the focus groups. It was argued that, as relatives, they had to accept undignified and missed care, since this was due to political decisions that the health personnel could not influence. These explanations were reinforced by quotes from health personnel employed at the nursing homes, who had told the relatives about shortages, fatigue, and how they experienced not being able to provide sufficient care. Because of this information, relatives described being familiar with both nonconformance reports and sick reports from health personnel related to stress.

Implicit in these descriptions was also an understanding that health personnel, similar to relatives, suffer when they have to accept unacceptable situations linked to the residents’ dignity. This cohesion also comes into view when the relatives referred to conversations where health personnel breached the duty of confidentiality, as participant 3, a daughter, explained in focus group 4:

*…yes, but I am a little shocked when those who work here say all these things to us relatives. Yes, there is plenty of it, it is a lot, and they plump out with things like what you described now. Ugh...*

In contrast to the explanations for external causes of indignity, several participants claimed that they did not accept that a lack of time or health personnel was always the reason for observed indignity. Differences in attitudes and competence among health personnel were suggested as alternative explanations. These examples included observations of how the residents could eat well if they had enough time and facilitation but that this varied depending on the individual health personnel at work at the particular time. Quality variations were also described between the two different nursing homes and between the different units in nursing home B, which the relatives described to have the lowest quality.

However, the participants revealed that they commonly made a conscious decision not to bring these conditions to the attention of the health personnel or the middle manager. They justified this choice based on past experiences where their feedback did not lead to any meaningful change and that they feared the potential consequences of becoming unpopular. They even expressed concerns that raising these issues could have a negative impact on the resident when the relative was not present. Instead, the relatives internalized their suffering as a mixture of anger and sadness based on an experience of cross-pressure of numerous conflicting perspectives based on individual values, family resources, institutional policies and the individual values of health personnel. The relationship between these perspectives and Galvin and Todres’ theoretical framework^10^ will be further elaborated in the discussion.

## Discussion

The aim of this study was to provide a critical discussion of relatives’ experiences of suffering when their next of kin live in a nursing home. The findings that form the main theme, suffering while resigning to an unacceptable violation of dignity, add a critical contrast to the knowledge we have about how close interaction with relatives promotes the quality of care results in nursing homes.^2,3,9^ The 18 participating relatives contributed critical reflections in five focus groups. Particularly by identifying how shortcomings in the nursing home interaction influence relatives to be reluctant to report deviations and suggestions for improvement. As a contribution to how Puurveen et al.^8^ describe the relationships between relatives and residents in nursing homes to be rooted in family-resident relationships, influenced by family-health personnel relationships and wider sociopolitical factors, our study demonstrates how challenging this area can be experienced.

The findings in this study are consistent with previous knowledge, when relatives describe continuing as central caregivers after their next of kin have entered a nursing home, and how this relates mostly to spouses and female relatives.^2^ Our findings add to this knowledge when describing relatives in rural areas as actively present in nursing homes. However, the first subtheme, suffering while adapting to a relationship of dependence, shows this to be a dependent relationship, where relatives experience suffering when they attempt to adapt to contrasting needs. The second subtheme, suffering while accepting the unacceptable, shows how relatives’ presence in nursing homes implies a conflicting process whereby relatives observe indignity and missed care without experiencing an ability to influence this situation.

**Figure 1.**
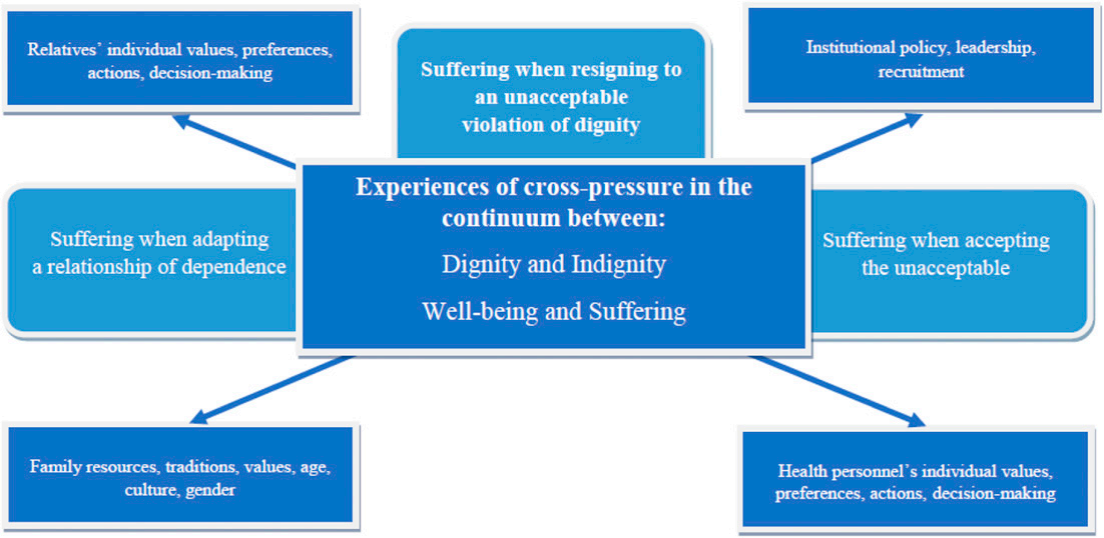
Relatives’ experiences of cross-pressure in the continuum between dignity and indignity and between well-being and suffering.

In our previous study,^11^ relatives’ experiences of suffering were found within all six domains as described by Galvin and Todres.^10^ In this study, relatives’ narratives add a number of examples of experienced suffering that we sum up as cross-pressure and that cannot easily be incorporated into Galvin and Todres’^10^ framework. Our starting point in Galvin and Todres’^10^ descriptions is thus expanded with a new perspective. The relationship between this study’s experienced cross-pressure and Galvin and Todres’ framework^10^ is depicted in Figure 1.

Figure 1 illustrates how the experiences of cross-pressure relate to the relatives’ perception of the residents’ lifeworld.^23^ Supporting the theoretical framework developed by Galvin and Todres,^10,12^ the main theme, suffering while resigning to an unacceptable violation of dignity, is formed by the relatives’ experience of cross-pressure in the continuum between the residents’ dignity and indignity,^12^ as well as between their well-being and suffering.^10^

The first subtheme, suffering while adapting to a relationship of dependence, is visualized on the left side of the figure as cross-pressure between relatives’ individual values, preferences, actions, and decision-making. This is in contrast to family resources, traditions, values, age, culture, and gender. The second subtheme, suffering while accepting the unacceptable, is shown on the right side of the figure as cross-pressure between institutional policy, leadership, and recruitment, in contrast to health personnel’s individual values, preferences, actions, and decision-making. The cross-pressure between the right and left sides of this figure is visualized as two axes (arrows). The first axis refers to the cross-pressure between relatives and health personnel’s individual values, preferences, actions, and decision-making. The second axis shows the cross-pressure between family resources, traditions, values, age, culture, and gender, as a contrast to the nursing homes’ institutional policy, leadership, and recruitment.

When adding cross-pressure as a new perspective to Galvin and Todres’^10^ framework of dignity, well-being, and suffering, this study builds on how the relatives’ narratives add a number of examples of experienced suffering that are incorporated in each other. One example of such a narrative is described when relatives cite insecurity and sorrow as central emotions when their next of kin enter the nursing home. These are perspectives on suffering that can be linked both to temporality and identity.^10^ This study substantiates Galvin and Todres^10^ descriptions of suffering in the continuum of three forms of spatiality, when the shared experiences of relatives describe meetings with health personnel who prefer relatives to stay in the resident’s room during visits, and how this is interpreted as them being banished from the communal areas. These are strong descriptions, where suffering as a result of spatiality^10^ can be understood as giving the relatives honor wounds^12^ related to the stigma of not belonging. The participating relatives describe suffering as linked to a general feeling of not belonging in the nursing home and being trapped by the interpretation of what they experience as rigid individually interpreted institutional rules. In these experiences, perspectives on suffering a loss of their interpersonal dignity are added to the cross-pressure, consistent with both spatiality and intersubjectivity.^10^ The findings also show how negative feelings that could be linked to mood^10^ appear when relatives experience incomprehensible power being used by health personnel, as exemplified when being asked to stay in the residents’ room.

The descriptions of suffering when observing missed care in this study can be related to all the levels and forms of suffering, as elaborated by Galvin and Todres.^10^ Despite relatives’ descriptions of how they monitor the residents’ embodied and interpersonal dignity, they mainly choose to take a passive role in the nursing home context, which conforms to institutional norms. However, this leads to an experience of overall suffering, a cross-pressure comprising a spectrum of perspectives, with several contemporaneous levels and forms. Based on these findings, suffering could be perceived as a cross-pressure when relatives experience being alienated in the nursing home’s everyday life. The sense of powerlessness, distrust, and guilt experienced by relatives, as described by Jakobsen et al.,^4^ therefore all become terms that are too mild in this pressure cooker of mixed emotions described by relatives in this current study. Where other studies describe how dignified care is expected by relatives,^6^ the findings in this study reveal an inherent expectation that this is no longer possible to achieve.

The participating relatives relate the missed care to external factors that health personnel cannot influence. These findings support Gustafsson et al.’s^14^ description of missed care as a result of the imbalance between patients’ need for nursing care and available resources, making this an ethical issue that challenges nurses’ professional and moral values. However, this differs from the findings by, among others, Lindwall and Lohne,^15^ who provide descriptions of missed care that are more closely related to the characteristics and shortcomings of health personnel. This study adds to this knowledge with examples of missed care as a result of the varying competence and individual characteristics of health personnel. However, the relatives’ critical reflection in the focus groups did not lead to any attacks on individuals; in contrast, there was a strong focus on protecting the health personnel.

The observations from relatives in this study support the descriptions of missed care from The Francis Report^13^ and add knowledge that contributes to an understanding that missed care is not context-bound, since the same experiences are described in a rural Norwegian context as in the UK. Several studies highlight how the complexity of care needs increases the risk of indignity in nursing homes.^20,21^ This is confirmed by the findings of this study, where it is the nursing home that has the most complex group of residents, in terms of diagnosis and functional impairment, that relatives experience as providing the greatest risk of indignity. The findings also support the descriptions of how a loss of relatives to support their need for care and social interaction, can increase the risk of missed care in nursing homes.^21^

### Strengths and limitations

Adding to Galvin and Todres’^10^ existential theoretical framework, which describes a continuum between well-being and suffering, this study reveals the main characteristics of suffering as a result of cross-pressure. However, this is to be understood as an initial overall description, knowing that it is necessary to build on this with extended knowledge.

This study has its strengths, as it is conducted in a rural sociodemographic context, which is considered an underexplored area of research in this field.^2^ Giving relatives the opportunity to come together and share their experience from the nursing home contexts was pre-understood in this study to provide important knowledge while at the same time empowering the relatives. This preunderstanding was confirmed when the focus group participants repeatedly expressed how no one had previously asked them about their experiences, and that they felt they had much to contribute when it came to improving practice. The participants also emphasized that they felt strengthened by the support of the other focus group participants, as they had shared experiences.

However, out of a total of 95 residents, only 16 residents were represented by 18 participants in this study. On the other hand, relatives are a group we know to be under pressure and in psychological distress.^35^ The relatives who chose to participate in this study were particularly involved in the nursing home, and most were present daily, while some visited several times a day, and others weekly.

This study solely involves limited data from a specific context in two Norwegian nursing homes, and the findings cannot be generalized to other contexts. However, our study had more contextual similarities than differences compared to published studies of other international nursing home contexts. It is also possible to consider the findings as “indicative” or transferable to other similar situations or settings.^32^

## Implications for policy, practice, and theory

The findings of this study indicate that there is a great distance from the health authorities’ guidance on the involvement of relatives in nursing homes to the relatives’ experienced reality. For relatives, the findings provide knowledge of how the strong feelings of suffering can be explained as a result of the cross-pressure they are under and can in this way become more manageable. For health personnel, the findings provide knowledge about relatives’ suffering, which may provide an opportunity for increased understanding of how this pressure cooker of emotions in some cases bursts. This knowledge is useful in designing interventions that increase opportunities for relatives to experience professional meetings with health personnel based on reflected choices and common goals of dignity in care. For managers and decision-makers, the knowledge from this study may increase opportunities to plan more targeted measures for quality improvement and may provide an opportunity to reduce the distance between government guidelines and practical everyday life in nursing homes.

## Conclusions

When their next of kin live in a nursing home, relatives experience suffering as cross-pressure. Cross-pressure is experienced when suffering occurs simultaneously in different forms and at different levels. Suffering is presented through the relatives’ experience of the residents’ lifeworlds, in a continuum between dignity and indignity, and between own or the resident’s well-being and suffering. These experiences comprise contrasts between, on the one hand, the institutional policy, the management, and the individual health personnel; on the other hand, the family relationship and the individual relatives who, in this cross-pressure, struggle in their attempts to interact responsibly with health personnel. The findings of this study describe how this struggle can have a negative outcome, where relatives end up resigning, trying to adapt to witnessing an unacceptable violation of dignity and undignified care, while the feeling of suffering builds up inside.

## Data Availability

The datasets used and/or analyzed during the current study are available from the corresponding author upon reasonable request.^36^
